# Mechanisms underlying elevated SBP differ with adiposity in young adults: the Enigma study

**DOI:** 10.1097/HJH.0000000000000798

**Published:** 2015-12-24

**Authors:** Jessica E. Middlemiss, Karen L. Miles, Barry J. McDonnell, Yasmin, Kaisa M. Maki-Petaja, John R. Cockcroft, Ian B. Wilkinson, Carmel M. McEniery

**Affiliations:** a Division of Experimental Medicine and Immunotherapeutics, University of Cambridge. Addenbrooke's Hospital, Cambridge; b Cardiff School of Health Sciences, Cardiff Metropolitan University; c Wales Heart Research Institute, Cardiff University, University Hospital, Cardiff, UK

**Keywords:** adiposity, BMI, cardiac output, peripheral vascular resistance, SBP

## Abstract

**Objectives:**

The positive association between adiposity and hypertension is well recognized. However, not all overweight individuals have elevated blood pressure (BP). Moreover, different factors may be associated with high BP in normal-weight versus overweight individuals. The aim of the current study was to examine the influence of adiposity on the relationship between SBP and underlying haemodynamic mechanisms in young adults.

**Method:**

Data from 2502 patients were available from the Enigma study. Detailed demographic, biochemical, and haemodynamic data were obtained in all individuals. Data were analysed between lower and upper tertiles of BMI and SBP, separately for each sex.

**Results:**

In normal-weight individuals, cardiac output (CO) was elevated in those with higher SBP, independently of body size. Moreover, higher CO was associated with an increased stroke volume in men (*P* < 0.001), but an increased heart rate in women (*P* = 0.002). In contrast, in overweight individuals, peripheral vascular resistance (PVR) was elevated in men with higher SBP (*P* = 0.02) and those with lower SBP had the lowest PVR of all groups. In linear regression analyses, there was a stronger association between SBP and CO in normal-weight individuals, but a stronger association between SBP and PVR in overweight individuals.

**Conclusion:**

Different haemodynamic mechanisms are associated with elevated SBP in young adults, depending on body size and sex. These data suggest the need for differential approaches to the identification and management of young adults with elevated BP.

## INTRODUCTION

Hypertension is a common condition and an important cause of morbidity and mortality worldwide [1]. Although hypertension is relatively rare in young adults, the prevalence of isolated systolic hypertension (ISH), the most common form of hypertension in the young [2–4], is increasing [5] and is associated with cardiovascular events in later life [6]. The underlying causes are probably because of multiple factors, with obesity, increased salt intake, and lack of physical activity all likely to play a significant role [7,8]. Indeed, the increased prevalence of ISH in the young observed in National Health and Nutrition Examination Survey III is thought to be because of obesity and smoking [5], both important risk factors for cardiovascular disease in later life.

Most studies report a positive association between blood pressure (BP) and obesity [9–11]. However, not all hypertensive patients are obese and not all obese individuals are hypertensive [8]. This suggests a variation in the effect of weight gain and that there may be adaptive or protective changes with regard to BP. It is also unclear as to whether the underlying pathophysiology differs between normal-weight and overweight hypertensives. Moreover, sex disparities in the natural history of hypertension have been examined [12–15], but the underlying haemodynamic mechanisms remain unclear.

We wished to examine the pathophysiology of BP elevation in young individuals, focusing on haemodynamic mechanisms and their relationship with weight and sex. We hypothesized that different haemodynamic mechanisms are responsible for elevated SBP in normal-weight versus overweight young adults and our aim was to test this hypothesis in a large population of young adults from the Enigma study.

## METHODS

Participants were drawn from the Enigma study population, which investigates the origins of hypertension with regard to clinical, physiological, and genetic characteristics [4]. Complete data were available in 2502 individuals, selected at random from two university populations in the United Kingdom (Cambridge and Wales). All individuals were aged between 18 and 40 years. Patients with secondary forms of hypertension or overt cardiovascular disease were excluded. Patients with diabetes mellitus, a serum cholesterol of 6.5 mmol/l, and/or renal disease were also excluded, as were patients receiving any vasoactive medication. The study was approved by the Local Research Ethics Committees, and all participants gave informed consent.

### Protocol

All participants completed a detailed lifestyle and medical history questionnaire and height, weight, and waist circumference were assessed and BMI was calculated. After 15 min of seated rest, brachial BP and radial artery waveforms were recorded. Following 20 min of supine rest, brachial BP and radial artery waveforms were reassessed, and pulse wave velocity (PWV) and cardiac output (CO) were determined, as described below.

Approximately 20 ml of blood was drawn from the antecubital fossa into plain tubes. The samples were centrifuged at 4°C (4000 rpm for 20 min) and the serum separated and stored at −80°C for subsequent analysis. Total cholesterol (TC), triglycerides, low-density lipoproteins (LDL), high-density lipoproteins (HDL) and glucose were assessed by standard automated biochemistry on a 2400 clinical chemistry system (Advia; Siemens Healthcare Diagnostics Inc, Newark, Delaware, USA) in an accredited laboratory. The LDL was calculated.

### Haemodynamics

Brachial BP was recorded in the dominant arm using appropriately sized cuffs and a validated oscillometric technique (HEM-705CP; Omron Corporation, Tokyo, Japan). Readings were taken in duplicate, or triplicate if readings differed by more than 5 mmHg. Radial artery waveforms were recorded with a high-fidelity micromanometer (SPC-301; Millar Instruments, Houston, Texas, USA) from the wrist of the dominant arm, and pulse wave analysis (SphygmoCor; AtCor Medical, West Ryde, New South Wales, Australia) used to generate a corresponding central (ascending aortic) waveform, as validated previously [16]. From this, central BP, augmentation index (AIx), augmentation pressure (AP), mean arterial pressure (MAP), and heart rate were calculated, as described previously [17]. Carotid–femoral (aortic) pulse wave velocity (aPWV) was recorded using the same device, as described previously [17]. In the subsequent analysis, augmentation pressure and augmentation index were adjusted for age, height, and heart rate, whereas aPWV was adjusted for age and MAP.

Cardiac output was assessed using a non-invasive, inert gas rebreathing technique, which has previously been validated against thermodilution and direct Fick methods for measurement of pulmonary blood flow and, thus, cardiac output [18–22]. Briefly, while resting, participants were instructed to continuously rebreathe a gas mixture (1% SF_6_, 5% N_2_0, and 94% O_2_) over 20 s, at a rate of 20 breaths/min. Expired gases were sampled continuously and analysed by an infrared photoacoustic gas analyser (Innocor; Innovision A/S, Odense, Denmark), for the determination of CO and stroke volume (SV). Peripheral vascular resistance (PVR) was calculated from the formula: PVR (dynes/s/cm^5^) = MAP (mmHg) × 80/CO (l/min). Trained investigators made all measurements. The within and between-observer measurement reproducibility values for the arterial stiffness and cardiac output measurements were in agreement with our previously published data [4,17].

### Statistical analysis

Data were analysed using Statistical Package for Social Sciences software (version 20.0; IBM, Armonk, New York, USA). The influence of BMI on SBP and the underlying haemodynamic mechanisms was examined using two approaches. Firstly, within each sex, participants were stratified into tertiles of SBP and BMI, with comparisons made between the upper and lower tertiles (i.e. the extremes), as an alternative to arbitrary thresholds, which might not be applicable to young adults. Independent samples *t*-tests and one-way analysis of variance were used to determine significant differences between the groups. Posthoc analyses were conducted using the Bonferroni method and Pearson χ^2^ method for categorical data. The independent samples, Kruskal–Wallis test was conducted for non-normally distributed data. Separately, linear regression models were constructed, treating all data as continuous variables, including terms for the interaction between BMI and haemodynamic factors (CO or PVR). All data are presented as means ± SD unless otherwise stated. The null hypothesis was rejected at *P* < 0.05.

## RESULTS

The demographics and basic haemodynamic characteristics of the study population are presented by sex in Table 1. Overall, CO was higher in men, and ISH was the most common form of hypertension. In contrast, PVR was higher in women and systolic diastolic hypertension was the most common form of hypertension.

### Biochemical characteristics

The demographic and biochemical characteristics are shown in Tables 2 and 3, for men and women, respectively. Applying the WHO criteria, participants in the lower BMI were categorized as ‘normal weight’ whereas those in the highest tertile were categorized as ‘overweight’. In both men and women, being overweight was associated with an adverse biochemical profile (higher TC, LDL triglycerides, glucose, and significantly lower high-density lipoproteins) compared with normal-weight individuals. This adverse profile was more pronounced in those participants who were overweight and had higher SBP.

### Haemodynamic characteristics

The haemodynamic characteristics are shown in Tables 4 and 5, for men and women, respectively. Those in the lower tertile of SBP were classified as having either optimal or normal BP, according to the recent European Society of Hypertension guidelines [23]. However, women in the upper tertile of SBP had either normal or high-normal BP, whereas men portrayed a predominantly ISH phenotype.

#### Normal-weight individuals

**FIGURE 1:**
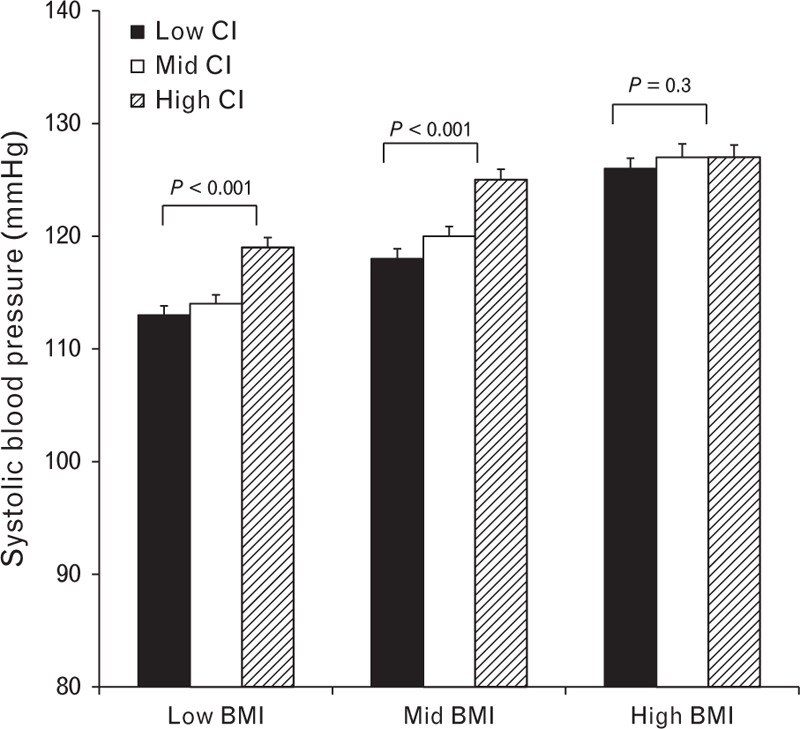
Influence of cardiac index on SBP in individuals in low, middle, and high tertiles of BMI. Data are means ± SEM.

In normal-weight individuals, CO was elevated in both men and women with higher SBP versus those with lower SBP. These differences remained after adjusting for differences in body size between the groups (Fig. 1). The higher CO in men was driven by a higher SV, which remained after adjusting for body size. In contrast, the higher CO in women was driven by a higher heart rate. There were no differences in other haemodynamic mechanisms between SBP groups in men. However, PVR adjusted for body size and augmentation pressure were elevated in women with higher SBP versus those with lower SBP. Higher brachial SBP was associated with a higher central SBP in both men and women and pulse pressure (PP) amplification did not differ between SBP groups in either men or women (Figure S1, Data Supplement).

#### Overweight individuals

In overweight individuals, CO was slightly higher in men and women with higher SBP versus those with lower SBP, but this trend was not significant, and adjusting for body size abolished any meaningful differences between SBP groups. However, in men, PVR was markedly higher in those with higher SBP, although this was not the case in women. In both men and women, PVR adjusted for body size was lowest of all in those with lower SBP. In women, higher SBP was associated with significantly higher augmentation pressure, AIx, and aPWV.

### Influence of BMI on the relationship between SBP, cardiac output, and peripheral vascular resistance

To examine further the influence of adiposity on the relationship between SBP, CO, and PVR, linear regression analyses were performed, using the entire cohort, treating the data as continuous variables. Overall, there was a significant, positive association between SBP and cardiac output in both men (*r* = 0.27, *P* < 0.001) and women (*r* = 0.25, *P* < 0.001). However, although similar associations were evident in normal-weight men (*r* = 0.31, *P* < 0.001) and women (*r* = 0.27, *P* < 0.001), the associations were much weaker in overweight men (*r* = 0.10, *P* < 0.05) and women (*r* = 0.12, *P* < 0.05). Indexing the cardiac output to body size did not alter the associations, as depicted graphically, using tertile analyses, in Figure 1.

**FIGURE 2:**
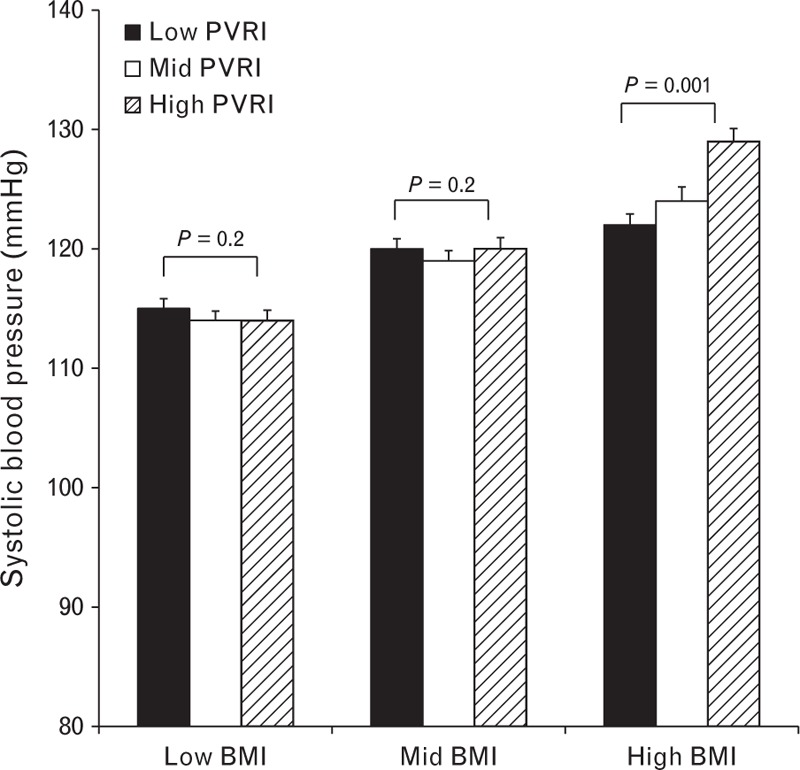
Influence of peripheral vascular resistance index on SBP in individuals in low, middle, and high tertiles of BMI. Data are means ± SEM.

There was a small, though significant association between SBP and PVR in women (*r* = 0.16, *P* < 0.001) overall, but not in men (*r* = 0.04, *P* = 0.2). However, there were stronger, positive associations between SBP and PVR in overweight men (*r* = 0.21, *P* < 0.001) and women (*r* = 0.28, *P* < 0.001), but not in normal-weight individuals (men: *r* = 0.02, *P* = 0.6; women: *r* = 0.1, *P* = 0.06). Again, adjusting for body size did not alter the associations (Fig. 2).

A series of stepwise multivariable regression models were then constructed in the same individuals to investigate whether the associations described above were independent of confounding factors. Both CO and PVR were independently associated with SBP, together with age, sex, and BMI (*R*^2^ = 0.56, *P* < 0.001, Supplementary Table 1). In addition, there were significant interactions between BMI and CO (model 2), PVR (model 3), and both CO and PVR (model 4), with the final model explaining 67% of the variance in SBP.

## DISCUSSION

We have investigated the relationship between elevated BMI and SBP with regard to haemodynamic and biochemical factors. As such, the current study provides a comprehensive examination of factors potentially underlying the association between adiposity and SBP in young adults. Our key findings were that increased CO was the predominant haemodynamic mechanism associated with higher SBP in normal-weight individuals. However, in overweight individuals, increased PVR, rather than CO, was associated with higher SBP, and overweight individuals with lower SBP had the lowest PVR of all groups. These data suggest that the mechanisms underlying increased SBP in young adults depend on body size, which may hold important implications for the treatment of high SBP in the young.

Previous haemodynamic studies in young adults have focused on individuals with either borderline [24] or sustained hypertension [25], and highlighted that an increased CO appears to be the key haemodynamic determinant of elevated BP in both normal-weight and obese individuals, albeit with different underlying mechanisms. In normal-weight participants, increased CO was thought to be associated with a hyperadrenergic state [26] whereas in obese individuals, adrenergic activity was thought to be normal but intravascular volume increased [27]. Although these earlier studies provided important insights into obesity-associated hypertension, the studies were relatively small and did not permit sex-specific analyses. Moreover, the average age of the study populations was ∼30 years and whether these patterns are evident in younger adults is unclear. Furthermore, CO was assessed using invasive methods, which may not be representative of the ‘usual’ resting state and may have elicited different stress responses between the different BP groups.

In the current study, we chose to examine haemodynamic characteristics across tertiles of SBP and in linear regression analyses, because these approaches did not rely on any arbitrary BP thresholds, which may not apply equally to men and women or simply to young adults *per se*. In addition, we examined CO using an inert gas rebreathing technique, which has been extensively validated to provide an accurate, non-invasive measurement of CO [18–22], without inducing stress responses typically seen with invasive measurements. In keeping with the studies mentioned above [24–27], we observed that elevated CO appeared to be the primary haemodynamic abnormality associated with elevated SBP in normal-weight individuals. However, we also noted that the raised CO was predominantly because of a high SV in men but high heart rate in women. To our knowledge, these are the first data describing such an effect of sex on the components of CO in young adults. As such, they confirm and extend our previous observations in young adults with ISH [4], which is almost exclusively confined to young men, largely because of the 140 mmHg threshold used to define the condition. Moreover, the current data highlight that different strategies may be needed to lower CO in men and women, if, indeed, this were considered a useful approach to lowering BP in young adults. Higher brachial SBP was also associated with a higher central SBP in both men and women. However, PP amplification did not differ between SBP groups in normal-weight individuals. Taken together, these observations argue strongly against any contention [28,29] that elevated SBP in young adults simply arises from exaggerated amplification of ‘normal’ central SBP.

We also observed that although CO was slightly higher in overweight, compared with normal-weight individuals, adjusting for body size abolished any differences between groups. Indeed, it is well recognized that CO, together with total blood volume, increases with body size [24,26,30,31]. Therefore, although elevated CO might be characteristic of overweight individuals with high SBP, this is likely to be because of secondary, passive effects of increased body size, rather than being a primary pathological mechanism. In contrast, elevated PVR appeared to be the primary haemodynamic abnormality associated with high SBP in overweight individuals. This was particularly apparent in linear regression analyses, where the association between PVR and SBP was significant in overweight, but not normal-weight individuals. Moreover, using the entire study sample, there was an independent, negative interaction between BMI and CO in association with SBP, but an independent, positive interaction between PVR and BMI indicating a stronger association between PVR and SBP with increasing adiposity. The interaction between BMI, CO, and PVR was strongest of all, with the model containing this term explaining the largest proportion of the variance in SBP, suggesting that adiposity has a significant influence on the interaction between CO and PVR in determining the level of SBP within an individual. Interestingly, PVR was lowest of all in overweight individuals with low SBP, especially after adjusting for body size. This observation highlights that an important adaptive mechanism may be present in at least some overweight individuals, which allows them to maintain lower levels of SBP despite their larger size. Such a mechanism may be related to structural or functional differences in the peripheral vasculature, either as a result of genetic predisposition and/or maintenance of a healthier obese phenotype [32] although clearly these hypotheses require testing in longitudinal studies. Nevertheless, the current data suggest that a greater understanding of the factors underlying adaptations in PVR is likely to provide important insights into the pathophysiology of hypertension in young adults.

Obesity and hypertension are frequently associated with metabolic abnormalities [33]. Previous data in young adults from the Tecumseh study [34] highlighted an adverse metabolic profile in individuals with borderline hypertension compared with those with normal BP. However, individuals were not stratified by BMI and those with borderline hypertension were more likely to be overweight (∼30%) than those with normal BP (∼13%). In the current study, we observed that a combination of high SBP and high BMI was associated with a worse biochemical profile, in terms of TC, and triglyceride levels, than with either factor studied in isolation. This was not altogether surprising, since risk factors tend to cluster, even in low-risk individuals [35]. However, the association with an adverse biochemical profile appears to depend more heavily on BMI rather than SBP, because stratification based on SBP alone revealed only incremental differences in biochemical profile between groups, whereas stratifying by BMI had a marked effect on biochemical profile, in both men and women.

Interestingly, higher alcohol consumption was evident in men and women with high SBP and there was a higher prevalence of smoking in overweight men with high SBP indicating that lifestyle factors may impact on the development of raised BP in young adults. Indeed, our data are in agreement with recent findings from National Health and Nutrition Examination Survey [5] demonstrating an increased prevalence of smoking in young adults with ISH.

More recently, the concept of early vascular ageing has received attention, particularly with regard to obesity, because early vascular changes are hypothesized to precede clinical manifestations of arterial hypertension in obesity, as reviewed by Jordan *et al.*[36]. In the current study, central SBP was highest in overweight individuals with increased brachial SBP, and PP amplification tended to be lowest in these individuals, suggestive of a higher central SBP for a given level of a brachial BP. In contrast, however, aPWV, a key biomarker of the early vascular ageing syndrome [37], was only elevated in overweight women with high SBP, after adjustment for the level of BP. As highlighted recently [36], the literature concerning the association between adiposity and aortic stiffness is yet to reach a consensus and further studies, with appropriate controls for confounding factors, are required.

### Limitations

There are a number of limitations of our study. Cross-sectional analyses do not permit causality to be examined and further longitudinal studies of weight gain and loss are required to test specific hypotheses generated from this analysis. In addition, our stratification was based, in part, on BMI, which is only a surrogate measure of body fatness and does not provide an accurate measure of body composition, particularly in highly muscular individuals [38]. Nevertheless, BMI is the most frequently used diagnostic tool in the classification of overweight and obesity. Lastly, we cannot exclude the possibility of an increased white-coat effect amongst the overweight individuals with high SBP, as reported previously [39,40], although every effort was made to ensure a quiet environment and standardized measurement conditions for all study participants. Ambulatory BP monitoring would be desirable in future studies.

### Clinical implications

Sustained essential hypertension is irreversible and remains a major risk factor for cardiovascular events. Moreover, recent evidence from younger and middle-aged adults demonstrates that ISH is associated with increased long-term risk for cardiovascular mortality compared with those with optimal or normal BP [6]. Therefore, understanding the mechanisms associated with early increases in BP is an important aim of strategies designed to identify those at risk of becoming hypertensive and/or preventing the longer-term development of the condition. We have demonstrated that the mechanisms underlying elevated SBP in young adults differs according to adiposity, with CO being the key abnormality in normal-weight individuals, and PVR being key in overweight individuals. This has important implications for the management of high SBP in young adults because existing therapies, which reduce CO, such as β-blockers, may be more useful in normal-weight young patients, whereas peripheral vasodilators may be more appropriate in overweight or obese patients, although clearly, further studies are required. Nevertheless, if targeting therapies toward the underlying abnormality is thought to be a useful strategy in retarding the development of hypertension, then choice of therapy may be dictated by body size.

## ACKNOWLEDGEMENTS

The Enigma study investigators: Samantha Benedict, J.C., Zahid Dhakam, Lisa Day, Stacey Hickson, K.M., B.M., C.M., J.M., K.M., Maggie Munnery, Pawan Pusalkar, Christopher Retallick, Ramsey Sabit, James Sharman, Jane Smith, Jean Woodcock-Smith, Edna Thomas, Sharon Wallace, I.W., Yasmin.

This work was funded by the British Heart Foundation and the National Institute for Health Research (NIHR) Cambridge Biomedical Research Centre.

### Conflicts of interest

There are no conflicts of interest.

## Supplementary Material

Supplemental Digital Content

## Figures and Tables

**TABLE 1:** Demographic and haemodynamic characteristics of the whole study population, by sex

Parameter	Men *n* = 1255	Women *n* = 1247	Overall *P*
Age (years)	23 ± 6	23 ± 6	0.04
Height (m)	1.79 ± 0.07	1.66 ± 0.07	<0.001
Weight (kg)	78 ± 14	64 ± 12	<0.001
Body mass index (kg/m^2^)	24.3 ± 3.9	23.2 ± 4.1	<0.001
Systolic blood pressure (mmHg)	128 ± 14	114 ± 14	<0.001
Diastolic blood pressure (mmHg)	77 ± 11	74 ± 11	<0.001
Cardiac output (l/min)	8.4 ± 2.1	6.5 ± 1.5	<0.001
Heart rate (beats/min)	72 ± 12	75 ± 12	<0.001
Peripheral vascular resistance (dynes/s/cm^5^)	881 ± 243	1066 ± 281	<0.001
Systolic diastolic hypertension (%)	8.8	4.3	<0.001^*^
Isolated systolic hypertension (%)	12.0	1.4	<0.001^*^
Isolated diastolic hypertension (%)	3.8	4.2	0.68^*^

Data are means ± SD.

^*^Pearson χ^2^.

**TABLE 2:** Demographic and biochemical characteristics in men, according to level of SBP and BMI

	Normal weight		Overweight		
Characteristic	Lower SBP *n* = 210	Higher SBP *n* = 68	Lower SBP *n* = 88	Higher SBP *n* = 212	Overall *P*
Age (years)	22 ± 5	22 ± 5^†^	25 ± 8^*^	26 ± 7^*^	<0.001#
Height (m)	1.79 ± 0.07	1.81 ± 0.07	1.78 ± 0.07	1.79 ± 0.06	0.07
Weight (kg)	65 ± 7	68 ± 7^†^	90 ± 12^*^	92 ± 12^*^	<0.001
BMI (kg/m^2^)	20.3 ± 1.4	20.8 ± 1.1^†^	28.3 ± 2.8^*^	28.8 ± 2.96^*^	<0.001
Family history of hypertension (%)	23	38	36	47^*^	<0.001#
Regular exercise (yes %)	81	86	78	80	0.6#
Alcohol (units/week)	11 ± 11	13 ± 12	13 ± 13	14 ± 13^*^	0.04#
Current smoker (%)	10	15	14	16	0.08#
Waist circumference (cm)	74 ± 5	76 ± 4†	93 ± 9^*^	92 ± 8^*^	<0.001
TC (mmol/l)	3.74 ± 0.8	4.05 ± 1.3^†^	4.20 ± 0.9^*^	4.63 ± 1.10^*^^,^^‡^	<0.001
LDL (mmol/l)	2.01 ± 0.68	2.23 ± 1.2^†^	2.4 ± 0.8^*^	2.65 ± 1.02^*^	<0.001
HDL (mmol/l)	1.37 ± 0.32	1.41 ± 0.37^†^	1.21 ± 0.2^*^	1.21 ± 0.29^*^	<0.001
Triglycerides (mmol/l)	0.89 ± 0.56	1.0 ± 0.52^†^	1.31 ± 0.8^*^	1.75 ± 1.33^*^^,^^‡^	<0.001
Glucose (mmol/l)	4.67 ± 0.79	4.83 ± 1.0	4.96 ± 1.26	5.02 ± 0.76^*^	0.02

Data are means ± SD. #Refers to non-normally distributed data. BMI, body mass index; HDL, high-density lipoproteins; LDL, low-density lipoproteins; TC, total cholesterol.

^*^P < 0.05 versus normal-weight, lower SBP.

^†^*P* < 0.05 normal-weight, higher SBP versus overweight, higher SBP.

^‡^*P* < 0.05 overweight, lower SBP versus overweight higher SBP.

**TABLE 3:** Demographics and biochemical characteristics in women, according to level of SBP and BMI

	Normal weight		Overweight		
Characteristic	Lower SBP *n* = 197	Higher SBP *n* = 81	Lower SBP *n* = 91	Higher SBP *n* = 189	Overall *P*
Age (years)	22 ± 4	24 ± 6	22 ± 6	25 ± 7^*^^,^^‡^	<0.001#
Height (m)	1.65 ± 0.06	1.66 ± 0.07	1.65 ± 0.07	1.65 ± 0.07	0.5
Weight (kg)	53 ± 5	56 ± 5^†^	73 ± 11^*^	77 ± 13^*^^,^^‡^	<0.001
BMI (kg/m^2^)	19.4 ± 1.2	20.1 ± 0.7^†^	26.73 ± 3.6^*^	28.10 ± 4.2^*^^,^^‡^	<0.001
Family history of hypertension (%)	24	35	29	51^*^^,^^‡^	<0.001#
Regular exercise (yes)	81	90	78	76	0.06#
Alcohol (units/week)	5 ± 5	7 ± 9^*^	8 ± 6^*^	8 ± 7^*^	<0.001#
Current smoker (%)	5	10	15^*^	13^*^	0.02#
Waist circumference (cm)	68.1 ± 7.2	69.0 ± 5.2^†^	81.5 ± 9.2^*^	84.3 ± 10.5^*^	<0.001
TC (mmol/l)	4.15 ± 0.9	4.37 ± 0.8	4.25 ± 0.82	4.54 ± 0.95^*^	<0.001
LDL (mmol/l)	2.30 ± 0.8	2.34 ± 0.7^*^^,^^†^	2.36 ± 0.76	2.58 ± 0.82^*^	0.009
HDL (mmol/l)	1.56 ± 0.4	1.67 ± 0.4^†^	1.46 ± 0.3	1.46 ± 0.4	<0.001
Triglycerides (mmol/l)	0.78 ± 0.4	0.89 ± 0.4^†^	1.05 ± 0.7^*^	1.20 ± 0.8^*^	<0.001
Glucose (mmol/l)	4.41 ± 0.8	4.64 ± 0.88	4.72 ± 0.7^*^	4.67 ± 0.6^*^	0.002

Data are means ± SD. #Represents independent samples Kruskal–Wallis test for non-normally distributed data. BMI, body mass index; HDL, high-density lipoproteins; LDL, low-density lipoproteins; TC, total cholesterol.

^*^P < 0.05 versus normal-weight, lower SBP.

^†^*P* < 0.05 normal-weight, higher SBP versus overweight, higher SBP.

^‡^*P* < 0.05 overweight, lower SBP versus overweight, higher SBP.

**TABLE 4:** Haemodynamic characteristics in men

	Normal weight		Overweight		
Characteristic	Lower SBP *n* = 210	Higher SBP *n* = 66	Lower SBP *n* = 87	Higher SBP *n* = 199	Overall *P*
Brachial SBP (mmHg)	113 ± 7	142 ± 8^*^^,^^†^	115 ± 5	146 ± 11^*^^,^^‡^	<0.001
Brachial DBP (mmHg)	70 ± 6	82 ± 10^*^^,^^†^	75 ± 8^*^	88 ± 11^*^^,^^‡^	<0.001
Central SBP (mmHg)	96 ± 6	109 ± 10^*^^,^^†^	100 ± 7^*^	116 ± 12^*,‡^	<0.001
PP amplification (ratio)	1.64 ± 0.13	1.68 ± 0.13^†^	1.56 ± 0.14^*^	1.62 ± 0.16^‡^	<0.001
MAP (mmHg)	83 ± 6	99 ± 10^*^^,^^†^	87 ± 7^*^	105 ± 10^*^^,^^‡^	<0.001
Heart rate (beats/min)	72 ± 12	74 ± 12	70 ± 10	75 ± 13^‡^	0.002
Cardiac output (l/min)	7.5 ± 2.1	9.3 ± 2.7^*^	8.5. ± 2.2^*^	8.9 ± 2.0^*^	<0.001
Cardiac index (l/min per m^2^)	4.1 ± 1.0	4.9 ± 1.3^*^^,^^†^	4.0 ± 1.0	4.2 ± 0.9	<0.001
Stroke volume (ml)	99 ± 32	117 ± 41^*^	116 ± 29^*^	115 ± 30^*^	<0.001
Stroke volume index (ml/m^2^)	54 ± 16	62 ± 21^*^^,^^†^	55 ± 13	55 ± 14	0.006
PVR (dynes/s/cm^5^)	904 ± 255	908 ± 343	799 ± 222	926 ± 245^‡^	0.02
PVRI (dynes/s/cm^5^/m^2^)	506 ± 163	487 ± 197	385 ± 117^*^	445 ± 130^*^	<0.001
Adjusted AP (mmHg)	0.6 ± 3.9	0.6 ± 3.8	0.2 ± 3.8	0.8 ± 3.9	0.09
Adjusted AIx (%)	1.4 ± 10.9	−1.9 ± 10.6	0.9 ± 10.7	1.5 ± 10.9	0.16
Adjusted a PWV (m/s)	5.93 ± 0.86	5.95 ± 0.81	6.11 ± 0.82	6.13 ± 0.97	0.1

Data are means ± SD. Aortic pulse wave velocity adjusted for age and mean arterial pressure; augmentation pressure and augmentation index adjusted for age, heart rate, and height. AP, augmentation pressure; AIx, augmentation index; MAP, mean arterial pressure; PP, pulse pressure; PVR, peripheral vascular resistance; PVRI, Peripheral vascular resistance index.

^*^P < 0.05 versus normal-weight, lower SBP.

^†^*P* < 0.05 normal-weight, higher SBP versus overweight, higher SBP.

^‡^*P* < 0.05 overweight, lower SBP versus overweight, higher SBP.

**TABLE 5:** Haemodynamic characteristics in women

	Normal weight		Overweight		
Characteristic	Lower SBP *n* = 197	Higher SBP *n* = 79	Lower SBP *n* = 91	Higher SBP *n* = 173	Overall *P*
Brachial SBP (mmHg)	100 ± 6	126 ± 9^*^^,^^†^	102 ± 4	130 ± 12^*^^,^^‡^	<0.001
Brachial DBP (mmHg)	67 ± 6	82 ± 9^*^	69 ± 6	84 ± 11^*^^,^^‡^	<0.001
Central SBP (mmHg)	87 ± 7	105 ± 12^†^	91 ± 7^*^	109 ± 14^*^^,^^‡^	<0.001
PP amplification (ratio)	1.59 ± 0.17	1.58 ± 0.21	1.58 ± 0.18	1.54 ± 0.2^*^	0.03
MAP (mmHg)	77 ± 6	97 ± 9^*^	80 ± 5	100 ± 11^*^^,^^‡^	<0.001
Heart rate (beats/min)	72 ± 12	78 ± 13^*^	73 ± 11	76 ± 12^*^	<0.002
Cardiac output (l/min)	5.8 ± 1.4	6.6 ± 1.6^*^	6.5 ± 1.5^*^	7.0 ± 1.7^*^	<0.001
Cardiac index (l/min per m^2^)	3.6 ± 0.9	4.1 ± 0.99^*^	3.6 ± 0.7	3.8 ± 0.9	<0.001
Stroke volume (ml)	80 ± 21	79 ± 23^†^	85 ± 21	88 ± 23^*^	<0.003
Stroke volume index (ml/m^2^)	51 ± 12	49 ± 13	47 ± 11	48 ± 12	<0.04
PVR (dynes/s/cm^5^)	1106 ± 280	1176 ± 390	1018 ± 263	1117 ± 323	0.17
PVRI (dynes/s/cm^5^/m^2^)	710 ± 198	737 ± 261^†^	573 ± 172^*^	619 ± 204^*^	<0.001
Adjusted AP (mmHg)	1.6 ± 3.6	3.0 ± 3.6^*^	1.7 ± 3.6	3.6 ± 3.6^*^^,^^‡^	<0.001
Adjusted AIx (%)	5.7 ± 11.7	8.8 ± 11.5	6.1 ± 11.4	10.2 ± 11.6^*^^,^^‡^	0.003
Adjusted PWV (m/s)	5.49 ± 0.84	5.71 ± 0.78	5.64 ± 0.76	5.79 ± 0.90^*^	0.04

Data are means ± SD. Aortic pulse wave velocity adjusted for age and mean arterial pressure; augmentation pressure and augmentation index adjusted for age, heart rate, and height. AP, augmentation pressure; AIx, augmentation index; MAP, mean arterial pressure; PP, pulse pressure; PVR, peripheral vascular resistance; PVRI, Peripheral vascular resistance index.

^*^P < 0.05 versus normal-weight, lower SBP.

^†^*P* < 0.05 normal-weight, higher SBP versus overweight, higher SBP.

^‡^*P* < 0.05 overweight, lower SBP versus overweight, higher SBP.
