# Supplementary material for: Mechanisms underlying elevated SBP differ with adiposity in young adults: the Enigma study
Source: J Hypertens. 2015 Dec 24;34(2):290–7. doi: 10.1097/HJH.0000000000000798 (PMC4697956; doi:10.1097/HJH.0000000000000798)
Supplement: Supplemental Digital Content [file jhype-34-290-s001.docx]

**Mechanisms underlying elevated systolic blood pressure differ with adiposity in young adults: The Enigma Study**

**Supplementary results**

Figure S1: Pulse pressure amplification in males and females with lower and higher SBP, respectively. Data are means±SEM.

Supplementary Table 1: Summary of Models 1-4

Supplementary Model 1

Supplementary Model 2

Supplementary Model 3

Supplementary Model 4

**Supplementary Figure 1.**

**Supplementary Table 1:**

| **SBP** | **Beta** | **R^2^** | ***P*** |
| --- | --- | --- | --- |
| Model 1:  Age, Gender, BMI, CO, PVR  Model 2:  Model 1 + BMI x CO  Model 3:  Model 1 + BMI x PVR  Model 4  Model 1 + BMI x CO x PVR | 0.09 | 0.55 | <0.001 |
|  |  |  |  |
|  | -0.41 | 0.55 | <0.001 |
|  | 0.78 | 0.56 | <0.001 |
|  | 1.09 | 0.67 | <0.001 |

**Supplementary Model 1:**

| **SBP** | **Beta** | **R^2^ change** | ***P*** |
| --- | --- | --- | --- |
| Adjusted R^2^ = 0.56 | | | |
| Age | 0.09 | 0.7 | <0.001 |
| Gender | -0.45 | 21 | <0.001 |
| BMI | 0.33 | 10.9 | <0.001 |
| CO | 0.19 | 2.9 | <0.001 |
| PVR | 0.78 | 19.6 | <0.001 |

**Supplementary Model 2:**

| **SBP** | **Beta** | **R^2^ change** | ***P*** |
| --- | --- | --- | --- |
| Adjusted R^2^ = 0.56 | | | |
| Age | 0.09 | 0.7 | <0.001 |
| Gender | -0.27 | 10.1 | <0.001 |
| BMI | 0.37 | 1 | <0.001 |
| CO | 1.22 | 5.3 | <0.001 |
| PVR | 0.78 | 12.6 | <0.001 |
| BMI X CO | -0.41 | 25.3 | <0.001 |

**Supplementary Model 3:**

| **SBP** | **Beta** | **R^2^ change** | ***P*** |
| --- | --- | --- | --- |
| Adjusted R^2^ = 0.56 | | | |
| Age | 0.08 | 0.5 | <0.001 |
| Gender | -0.28 | 21 | <0.001 |
| BMI | -0.23 | 10.9 | <0.001 |
| CO | 0.92 | 2.9 | <0.001 |
| PVR | 0.09 | - | 0.4 |
| BMI X PVR | 0.78 | 20.5 | <0.001 |

**Supplementary Model 4:**

| **SBP** | **Beta** | **R^2^ change** | ***P*** |
| --- | --- | --- | --- |
| Adjusted R^2^ = 0.67 | | | |
| Age | -0.004 | - | 0.79 |
| Gender | -0.27 | 13.2 | <0.001 |
| BMI | -0.67 | 11.4 | <0.001 |
| CO | 0.28 | 2.5 | <0.001 |
| PVR | 0.1 | 0.1 | <0.003 |
| BMI X CO X PVR | 1.09 | 40.1 | <0.001 |
